# Renal Function in Patients with Cystic Fibrosis: A Single-Center Study

**DOI:** 10.3390/ijerph19095454

**Published:** 2022-04-29

**Authors:** Marta Rachel, Sabina Galiniak, Marek Biesiadecki, Agnieszka Gala-Błądzińska

**Affiliations:** 1 Department of Allergology, Provincial Hospital No. 2, Lwowska 60, 35-301 Rzeszów, Poland; 2 Institute of Medical Sciences, Medical College of Rzeszów University, Rzeszów University, Warzywna 1a, 35-310 Rzeszów, Poland; mbiesiadecki@ur.edu.pl (M.B.); agala.edu@gmail.com (A.G.-B.); 3 Department of Internal Medicine, Nephrology and Endocrinology, Provincial Hospital No. 2, Lwowska 60, 35-301 Rzeszów, Poland

**Keywords:** cystic fibrosis, drug nephrotoxicity, kidney injury

## Abstract

Cystic fibrosis (CF) is the most common incurable autosomal recessive disease affecting the Caucasian population. As the prognosis for life extension of CF patients improves, co-morbidities, including kidney disease, become more common. Identifying those at the highest risk of kidney injury is therefore extremely important. The aim of this study was to evaluate the biomarkers of renal function in 50 CF patients using the estimated glomerular filtration rate (eGFR) based on creatinine and cystatin C equation as well as serum creatinine (sCr), serum cystatin C (CysC), serum urea and urinary neutrophil gelatinase-associated lipocalin (uNGAL) concentrations. sCr, CysC, urea and uNGAL were estimated. eGFR was calculated according to the CKD-EPI formula. CysC was significantly increased, while eGFR was significantly lower in the CF group than in the controls (*p* < 0.001 and *p* < 0.01, respectively). There was no significant difference in the sCr, urea and uNGAL concentrations between patients with CF and healthy subjects. For the purpose of our analysis, in order to assess renal function in patients with CF in clinical practice, the concentration of serum CysC and eGFR_CKD-EPI_ should be determined. Patients with CF presented with renal function impairment pictured by increased serum CysC and decreased eGFR values compared to controls. Unchanged uNGAL concentrations suggested preserved tubular function despite aminoglycoside treatment. Further prospective studies are needed to clarify whether kidney impairment observed in the course of CF progresses.

## 1. Introduction

Cystic fibrosis (CF) is a multi-system disease caused by mutations in a gene that leads to a defective or missing CF transmembrane conductance regulator (CFTR). In the Caucasian population, it is estimated to occur with an incidence of 1 in 2500 births, and about 1 person in 25 carries the defective gene [1,2]. CF is clinically characterized by chronic obstructive bronchial lesions and respiratory infections, as well as disturbed digestive processes and their consequences. Patients are often accompanied by pancreatic insufficiency, diabetes, malabsorption and liver problems. With the increase in survival time, technological advancement and improved quality of medical care, comorbidities have emerged [3]. Many complications that previously raised little concern have become more important, including kidney injury [4]. Renal function is not affected primarily in CF, but the kidney may be involved secondarily later. Pulmonary deterioration is the principal cause of CF-related mortality and morbidity. People with CF have an increased susceptibility to chronic lung infections, especially with *Pseudomonas aeruginosa*; therefore, aggressive antibacterial therapy is repeated. Most antibiotics used for treatment are administered intravenously and given for about two weeks [5,6]. People with CF are at risk of developing acute kidney injury (AKI) due to taking potentially nephrotoxic drugs, such as antibiotics, as well as long-term kidney injury caused by diabetes and systemic chronic inflammation, which may induce secondary amyloidosis with renal involvement [7,8]. In the literature, there are descriptions of CF associated with amyloidosis with renal involvement, nephrolithiasis, high-protein diet, secondary AKI due to dehydration, or nephrotoxic immunosuppressants, in the case of lung transplant recipients [6,9,10]. The possibility of kidney damage in patients with CF due to chronic heart failure resulting from pulmonary hypertension and chronic cardio–renal syndrome is also important [7,11]. An accurate assessment of renal function enables drug dosing and monitoring as well as early detection of kidney disease. Most commonly, assessments of renal function are limited to the measurement of the estimated glomerular filtration rate (eGFR) and the assessment of proteinuria [12]. Serum creatinine, the traditional biomarker of kidney impairment, is an unreliable marker of kidney function in patients with CF and reduced muscle mass [13]. For this reason, actual renal function in patients with CF is often overestimated and damage is not detected until a significant number of nephrons are affected. One biomarker of renal tubular injury is urinary neutrophil gelatinase-associated lipocalin (uNGAL), considered to be a biomarker of AKI [14]. Unfortunately, uNGAL may not always be a good biomarker in patients with CF as it is also formed in response to damaged epithelial cells in lungs [15]. Another biomarker used to assess renal function, independent of muscle mass, is serum cystatin C (CysC). For patients with CF who receive cycles of potentially nephrotoxic antibiotics to treat acute pulmonary exacerbations, evaluations of renal parameters are of particular importance. The aim of our study was to evaluate kidney function in patients with CF who received treatment in a local CF treatment center.

## 2. Materials and Methods

### 2.1. Study Population

We conducted a single-center, cross-sectional study to estimate renal functions in patients with CF between March 2019 and July 2020. Patients were recruited at the Department of Allergology and Cystic Fibrosis, Provincial Hospital No. 2 in Rzeszow. After informed consent was obtained, each subject participated in a screening visit that included a medical history review, a physical examination, spirometry, and serum and urine sampling. Nutritional status was assessed by the body mass index (BMI) value derived from the mass weight and height of a person in units of kg/m^2^. All patients underwent ultrasound examination of the kidneys. Patients received a specialized and recommended diet as well as vitamins, antibiotics, mucolytic drugs (Rh-Dnase) and pancreatic enzymes in case of pancreatic insufficiency. Pancreatic insufficiency was defined as a stool pancreatic elastase rate below 200 µg/g (58% of patients). In the study group, 4 people received inhaled Tobramycin in a dose of 300 mg twice a day for 3 years. Every 28-day cycle was followed by a 28-day break. Seven patients were treated with inhaled Colistin in a dose of 4 million units a day in cycles similar to Tobramycin.

### 2.2. Inclusion Criteria

The study involved patients with CF with a confirmed diagnosis based on the determination of sweat chloride, genetics and an immunoreactive trypsin test in neonatal age (patients born in or after 2009). The patients received nephrotoxic antibiotics a minimum of three times per year during their stay in a hospital after acute exacerbations. The average length of stay of patients in a hospital is presented in Table 1. The tests were performed each time after the antibiotic therapy during a hospital stay. The average dose of antibiotics depended on the patient’s weight and the antibiogram according to the prescribed standards.

All adult patients or volunteers gave their written informed consent to participate in the study. The consent for minors was given by parents or legal guardians.

### 2.3. Exclusion Criteria

Patients were excluded if they had heart failure, psychiatric disorder, post solid organ transplantation, urinary system pathology in ultrasonography and chronic renal failure. None of the patients met the AKI criteria of the Kidney Disease Improving Global Outcomes (KDIGO) [16]. They were excluded if they had not received antibiotics (non-chronically infected patients) and had received chronic oxygen or immunosuppressive therapy within 6 months prior to the study or if patients refused to participate.

**Figure 1:**
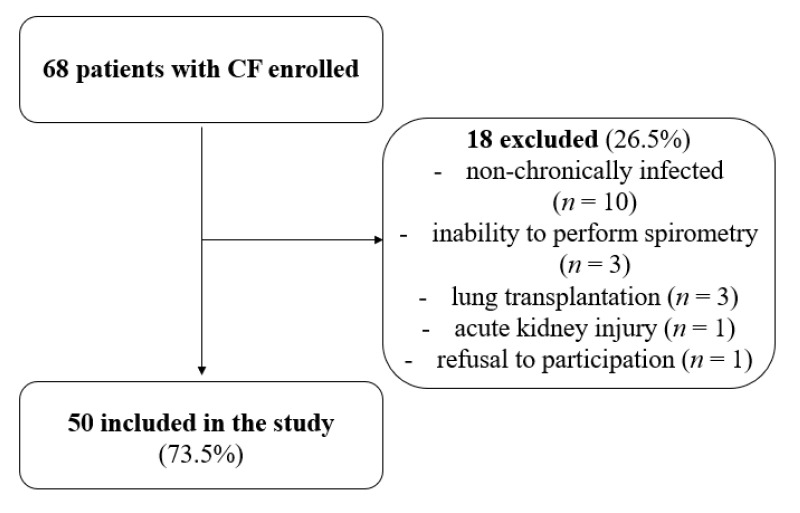
Flow chart of number of patients recruited and analyzed in the study.

Children up to 6 years of age, both healthy and those suffering from cystic fibrosis, were excluded from the study due to technical difficulties in performing a spirometry test. A flow chart outlining the recruitment process is shown in Figure 1.

Healthy subjects were recruited at the same time. The control group consisted of sex-matched volunteers without any disease, including urinary tract disease or infection in medical history and physical examination. The volunteers had not been taking any medications 30 days prior to the study.

### 2.4. Blood Collection

Blood samples were taken after an overnight fast. The results of blood counts were obtained using the hematology analyzer ADVIA2120i (Siemens Healthineers, Erlangen, Germany). The C-reactive protein (CRP) concentration was measured using the dry chemistry immunological method in a VITROS 250 analyzer (Ortho Clinical Diagnostics, Johnson and Johnson, Raritan, NJ, USA). Interleukin 6 (IL-6) was estimated using commercially available enzyme-linked immuno-sorbent assays (R&D Systems, Minneapolis, MN, USA) in line with the manufacturer’s instructions.

### 2.5. Urine Collection

The samples were collected from each participant by an appropriate method depending on their age. The normally chosen method was a clean catch urine sample into a sterile container.

### 2.6. Assessment of Renal Function

#### 2.6.1. Cystatin C (CysC)

CysC in serum was measured with a turbidimetric immuno-assay on Siemens Atellica SCI. The reference range for CysC is 0.64–1.23 mg/L.

#### 2.6.2. Estimating Glomerular Filtration Rate (eGFR)

eGFR was calculated according to the CKD-EPI formula based on creatinine. In addition, eGFR was calculated according to the chronic kidney disease epidemiology collaboration (CKD-EPI) cystatin C equation (2012) formula [17]. The normal level of eGFR is more than 90 mL/min/1.73 m^2^.

#### 2.6.3. Serum Creatinine (Cr)

Serum Cr was measured according to an enzymatic assay with creatininase and creatinase on Siemens Atellica SCI. The reference range for serum creatinine depends on the patient’s age and sex. For statistical compilation, the creatinine results were averaged and presented separately for patients under 18 years of age and adults.

#### 2.6.4. Serum Urea

Serum urea was measured using an enzymatic photometric assay with urease/GLDH on Siemens Atellica SCI. The reference range for serum urea is 19–49 mg/dL.

#### 2.6.5. Urine Neutrophil Gelatinase-Associated Lipocalin (uNGAL)

uNGAL was measured in the urine using a validated chemiluminescent microparticle immuno-assay on Abbott Architect i1000SR. The reference range for uNGAL is 0.0–131.7 ng/mL.

### 2.7. Sputum Collection

The agent used for sputum induction and collection was hypertonic saline, and deep throat swab specimens were cultured for bacterial pathogens. Bacterial and fungal colonizations/infections were classified on the basis of the criteria for *P. aeruginosa* infections, and those criteria were systematically applied to all bacterial/fungal species encompassed in this study [18]. The effect of the most frequently detected bacterial and fungal species (*P. aeruginosa*, *Staphylococcus aureus*, *Burkholderia cepacia*, *Stenotrophomona maltophilia* and *Candida albicans*) on lung function was analyzed. Chronic colonization by *P. aeruginosa* was defined by at least 3 positive sputum tests for that bacteria.

### 2.8. Spirometry

The severity of the respiratory system involvement (condition) was assessed on the basis of the forced expiratory volume within 1 s (FEV_1_, percentage of the predicted value) using a standard spirometry device (Lungtest 1000, MES, Kraków, Poland). Mean percent change in FEV1 was assessed for patients with CF and healthy subjects.

### 2.9. Data Analysis

Statistical analysis of the data was performed using the STATISTICA software package (version 13.1, StatSoft Inc. 2016, Tulsa, OK, USA). Data are presented as *n* (%), median and interquartile range as well as range. Normality of distribution was validated using the Shapiro–Wilk test, and skewness and kurtosis values, as well as on the basis of visual assessment of the histograms. Comparisons of the groups were conducted with the Mann–Whitney U test because of abnormality of the distribution. A *p*-Value < 0.05 indicates statistical significance. Spearman’s rank correlation coefficient analysis was employed to estimate the relationships between studied parameters, assuming linear dependence.

### 2.10. Ethics

The study was approved by the local Bioethical Commission (9/01/2020). All procedures performed in studies involving human participants were in accordance with the ethical standards of the institutional and/or national research committee and with the 1964 Helsinki declaration and its later amendments or comparable ethical standards. The study conformed to the principles outlined in the Declaration of Helsinki, and written consents were obtained from each patient with CF and with the volunteers who enrolled.

## 3. Results

Fifty patients with CF and fifty healthy volunteers were enrolled between March 2019 and July 2020. The demographic data and clinical characteristics are shown in Table 1.

**Figure 2:**
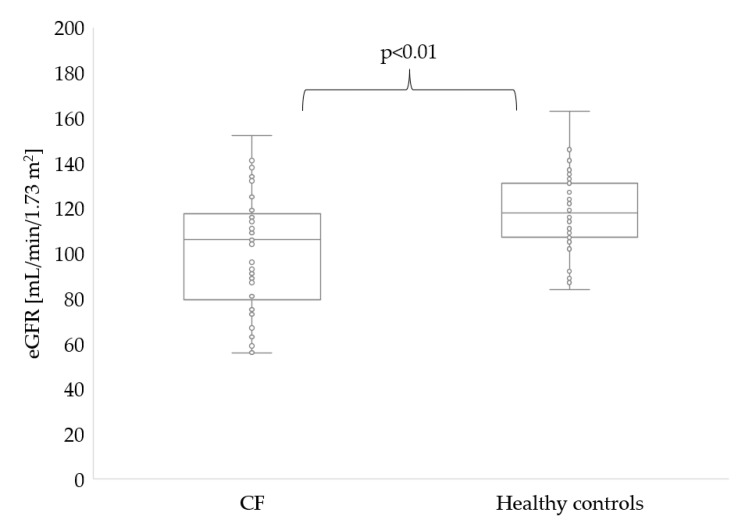
eGFR_CKD-EPI_ in patients with CF and healthy controls.

**Figure 3:**
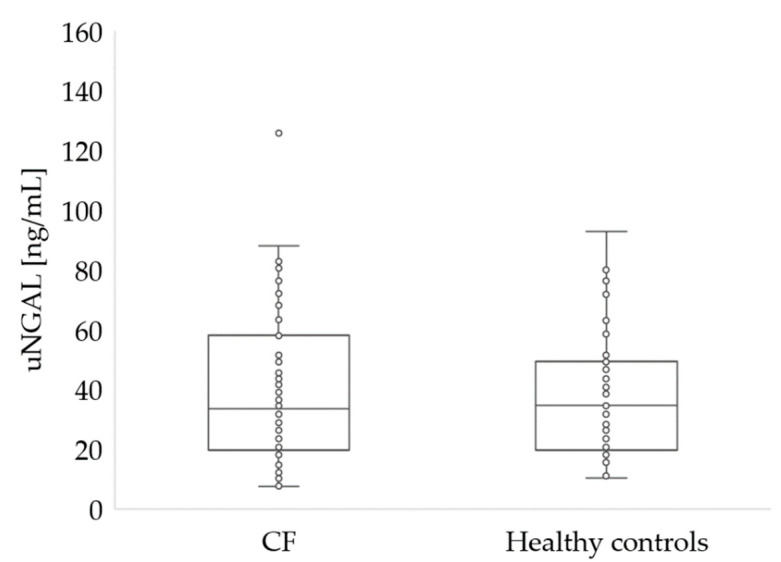
Urine NGAL concentration in patients with CF and healthy controls (*p* > 0.05).

The parameters characterizing the renal function of the study participants are summarized in Table 2 and in Figure 2 and Figure 3.

Significantly lower eGFR was noted in patients with CF (106 vs. 118 mL/min/1.73 m^2^, *p* = 0.002). In five patients with CF, eGFR level was lower than 80 mL/min/1.73 m^2^ (Figure 2).

There were no statistical differences in sCr levels between patients with CF and healthy controls (*p* = 0.576 for participants under 18 and *p* = 0.196 for adults). However, in 10 CF patients under 18 years of age, sCr levels were above normal. Blood urea level was similar in both groups (27 vs. 25, *p* = 0.097). However, in two patients with CF, the urea level was below normal, while seven patients with CF had urea levels above normal. No significant differences (*p* = 0.766) were noted in uNGAL concentrations in CF and healthy participants (Figure 3).

**Figure 4:**
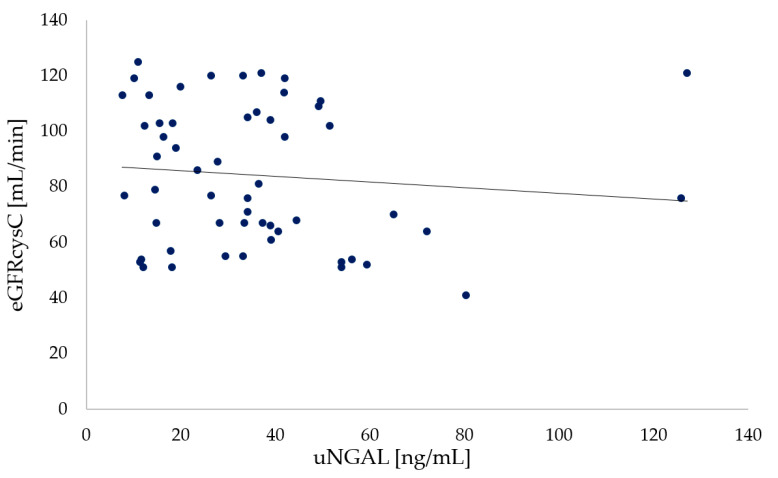
Relationship between urine NGAL and eGFRCKD-EPI Cystatin C in participants with CF (*p* = 0.272).

Similarly, no significant differences (*p* = 0.272) were noted between uNGAL concentrations and eGFR_CKD-EPI_ cystatin C values in participants with CF (Figure 4).

We noticed no statistical difference in the tested parameters between patients with and without CFRD (not shown). There were no abnormalities in the general urinalysis. Within the study group, no morphological changes in kidneys were shown in the ultrasound examination.

## 4. Discussion

Our study has shown that patients suffering from CF with chronic bronchial infection who in the previous year had received at least three courses of antibiotic therapy with potentially nephrotoxic drugs and stayed in the hospital at least 50 days have significantly higher serum cystatin C and lower eGFR_CKD-EPI_ values compared to healthy people. On the other hand, we have observed that biomarkers such as serum creatinine, serum urea and urinary NGAL cannot be used to assess renal function in patients with CF in clinical practice.

Accurate assessment of renal function is a prerequisite for the proper management of patients with CF at increased risk of renal failure due to CFTR genetics, amyloidosis, repeated treatment with aminoglycosides (associated with salt loss), fluid imbalance, diabetes, malnutrition, hypoxia and other clinical conditions. In contrast, the absence of an obvious renal phenotype in CF patients is a priori paradoxical because CFTR protein is expressed in the kidney, and it is involved in the regulation of many reabsorption and secretion mechanisms. One possibility to show that renal function in patients with CF is the replacement of CFTR function by other transporters or proteins where CFTR is absent or not functioning correctly. One candidate for this is TNR-CFTR, an alternative splice of CFTR, which, in vitro, functions as a wild-type CFTR [19]. The kidneys play an important role in sodium homeostasis. Under physiological conditions, they filter 25,000 mM sodium per day, of which they finally excrete 100–300 mM in the urine. The renal tubules have sodium reabsorption mechanisms that vary with the segment of the nephron. Aminoglycosides are examples of drugs that are implicated in tubular injury-related kidney injury. The tubular injury mainly affects the proximal tubular epithelial cells, particularly because of their metabolic load, the presence of various drug transporters, and their involvement in secretion and reabsorption of glomerular filtrates that exposes them to nephrotoxins. Damage to the renal tubules due to aminoglycosides results in impaired sodium reabsorption from primary urine and increased natriuria [20]. Additionally, aminoglycosides disrupt electrolyte homeostasis, leading to hypokalemia, hypomagnesaemia and hypocalcemia. It is suggested that aminoglycosides may alter the renal tubular function due to the mitochondrial dysfunction and an activation of the calcium-sensing receptor, resulting in excessive urinary chloride and calcium loss [21,22]. A known risk factor for renal function impairment including prior renal disease, acute dehydration or long-term treatment with a nephrotoxic drug was present in 75% of patients with CF and acute renal failure in the study by Smyth et al. [23]. Moreover, an increase in endothelial dysfunction and metabolic indexes in patients was related to reduced renal function, such as serum uric acid, triglycerides and LDL [10]. Additionally, Berg et al. proved that the incidence of chronic kidney disease (CKD) increased with age as well as in patients with diabetes, chronic infection, lung transplantation and aminoglycosides use [24]. The above-mentioned causes can significantly accelerate the disease-related decline in the glomerular filtration rate. Additionally, treating lung disease in CF may result in toxicity and drug–drug interaction complications, especially with aminoglycosides [25,26]. Renal pathologies in CF patients have been considered rare, but recently, renal problems such as diabetic glomerulopathy, amyloidosis, postinfectious glomerulonephritis, membranous nephropathy, chronic interstitial nephropathy or tubular damage have been observed more and more often [27]. The study by Muirhead et al. [28] revealed that AKI occurs in 19% of patients taking vancomycin and 9% of those taking tobramycin. It was shown that AKI was related to recent receipt of an aminoglycoside antibiotics, duration of therapy and low serum albumin. Interestingly, infection with *S. aureus* diminished the odds of developing AKI [29]. Moreover, people with AKI were at increased risk for long-term morbidity, mortality and the development of CKD [30]. The study conducted by Quon et al. [31] revealed that pulmonary exacerbations did not significantly increase the risk of CKD, whereas CF-related diabetes requiring insulin therapy substantially increased the risk of CKD. Accurate assessment of kidney function seems to be extremely important for patients with CF. The study conducted by Beringer et al. revealed that there were no significant differences in serum CysC between adults with CF compared to healthy volunteers (0.81 vs. 0.74 mg/L, *p* = 0.136) [32]. Moreover, they noticed that the use of GFR estimated from CysC might be an alternative marker with higher sensitivity and specificity than eGFR estimated from Cr (eGFR_MDRD_) in patients with CF [26,32]. In our study, the eGFR_CKD-EPI_ was decreased in CF group compared to healthy volunteers. The eGFR_CKD-EPI_ predicted by the Cr was higher than that predicted by serum CysC, similarly to the study by Hermida and Tutor [33]. In addition, serum Cr does not differ statistically significantly between CF and healthy volunteers. In the studies of other researchers, the conclusions regarding the clinical efficacy of Cr in people with CF are similar or different [10,32]. Therefore, we believe that the determination of Cr in patients with CF to assess kidney function is not a useful biomarker that is valuable in clinical practice.

In our study, the level of uNGAL did not differ between the patients with CF and healthy groups. Nevertheless, the patients with CF had significantly increased serum NGAL levels compared to the healthy subjects in the study by Zughaier et al. [34]. It was suspected that peripheral monocytes may contribute to high levels of serum NGAL in CF patients. Detailed studies revealed that monocytes from CF and healthy donors secrete similar amounts of NGAL. Furthermore, Zughaier et al. [34] noted no differences in serum NGAL levels between clinically stable patients with CF compared to participants undergoing pulmonary exacerbation. No increase in urinary NGAL detected in our study may be a result of damage of glomeruli in the CF population. In studies comparing that particular biomarker with others, it has been shown that the increase in uNGAL appears first; it is, therefore, an important biomarker of early detection of AKI [35,36]. Proteomic analysis confirmed that the NGAL protein was induced in the kidneys after ischemic and nephrotoxic AKI and that the concentration in urine increased several-fold in the early post-injury period. The total concentration of uNGAL in AKI probably represents a mixture of different molecular forms of NGAL with different cellular origins [37]. In theory, NGAL has promise as a biomarker of aminoglycoside-induced nephrotoxicity as it is taken up by proximal tubule epithelial cells via the same megalin-mediated endocytosis as aminoglycosides [38]. Notably, it has been demonstrated that in children and young adults with CF, significant changes occur acutely in uNGAL during exposure to tobramycin, which suggests that any biomarker with the potential for early detection of renal dysfunction should be assessed carefully before dismissing its utility in CF [39].

Recently, kidney injuries associated with CF have rapidly increased in number and developed more serious complications. Thus, it is important to pay attention to risk factors to eliminate complications and, in consequence, to improve the prognosis of CF patients. The predictors of kidney function impairment include treatment with aminoglycosides, diabetes and malnutrition. In addition, long term and average risk factors include a high-protein diet rich in oxalates or sodium chloride, and hypoxia [4,30]. Additionally, the emergence of a large number of comorbidities requires involvement of an increasing number of specialists in the treatment process, including nephrologists. Therefore, we believe that an assessment of kidney function in the course of CF is necessary and justified. The molecular mechanism requires further research to elucidate the pathway in more detail, and prospective studies with longer follow-up are warranted to investigate causation. Finally, early diagnosis and multidirectional treatment may be effective in inhibiting the progression of changes in the kidneys because scanty symptoms may delay diagnosis.

Interesting findings were pointed out in this report; however, several limitations of the study have to be mentioned. First, only patients with chronic bronchial infection and who in the previous year had received at least three courses of antibiotic therapy with potentially nephrotoxic drugs and had stayed in the hospital at least 50 days from one center were included in the study. Moreover, we did not assess the level of albuminuria in the urine. Additionally, while CysC is certainly a precise parameter for assessing kidney function, it is also expensive and difficult to apply to clinical inpatient practice. Additionally, in our study, there was lack of follow-up control of kidney function in order to perceive its potential decline with time, which is essential for those patients. Finally, aminoglycosides are not the first line of drugs for the treatment of CF patients with chronic infections.

## 5. Conclusions

CF patients presented with renal function impairment, pictured by increased serum CysC and decreased eGFR values compared to controls. Unchanged uNGAL concentrations suggested preserved tubular function despite aminoglycoside treatment. Further prospective studies are needed to clarify whether kidney impairment observed in the course of CF progresses.

## Figures and Tables

**Table 1:** Demographic and clinical characteristics of the studied groups *.

		CF	Healthy Controls	*p*
Age in years, median (Q1; Q3)		18 (14; 22)	17 (12.5; 20)	NS
	range	7–44	8–43	
	≥18 years, *n* (%)	29 (58%)	24 (48%)	NS
	<18 years, *n* (%)	21 (42%)	26 (52%)	NS
BMI, median (Q1; Q3)		18.4 (17; 21)	21.9 (20; 24)	<0.0001
	range	14–26	15–28	
Gender				
	female, *n* (%)	25 (50%)	27 (54%)	NS
	male, *n* (%)	25 (50%)	23 (46%)	NS
CF genotype				
	homozygote ΔF508, *n* (%)	33 (66%)	-	
	heterozygote ΔF508, *n* (%)	11 (22%)	-	
	other, *n* (%)	6 (12%)	-	
Colonization				
	*Pseudomonas aeruginosa*, *n* (%)	20 (40%)	-	
	*Staphylococcus aureus*, *n* (%)	25 (50%)	-	
	*Burkholderia cepacia*, *n* (%)	2 (4%)	-	
	*Stenotrophomonas maltophilia*, *n* (%)	3 (6%)	-	
	*Candida albicans*, *n* (%)	18 (36%)	-	
Average hospitalization time in days in the last year, median (Q1; Q3)		52 (50, 54)	-	
	range	42–62	-	
CF-related diabetes, *n* (%)		9 (18%)	-	
Acute exacerbation in last 6 months, *n* (%)		30 (60%)	-	
FEV_1_%, median (Q1; Q3)		78 (67; 88)	97.1 (90; 102)	<0.0001
	range	34–113	84–115	
WBC, * 10^3^/μL, median (Q1; Q3)		2.1 (0.5; 8.4)	1.5 (0.6; 2.3)	<0.0001
	range	0.1–32.9	0.2–4.8	
CRP, mg/L, median (Q1; Q3)		4.8 (3; 9.6)	1.7 (1.5; 2.5)	0.0234
	range		1.5–3.9	
IL-6, pg/mL, median (Q1; Q3)		9 (7.7; 11.8)	1.5 (0.6; 2.3)	<0.0001
	range	5–21.6	0.2–4.8	

* Data are shown as median and interquartile range as well as range or number of patients and percent; NS—not significant; abbreviations: BMI—body mass index; CF—cystic fibrosis; CRP—C-reactive protein; FEV1—first second of forced expiration; IL-6—interleukin 6; WBC—white blood cells.

**Table 2:** Parameters of renal function of the study participants *.

	Norm	CF (*n* = 50)		Healthy Controls (*n* = 50)		*p*
		Median (Q1; Q3)	Range	Median (Q1; Q3)	Range	
CysC [mg/L]	0.64–1.23	0.91 (0.818; 1)	0.7–1.16	0.78 (0.723; 0.868)	0.64–1.02	0.00003
sCr [mg/dL] < 18 years old	0.4–0.72	0.68 (0.56; 0.77)	0.43–0.99	0.65 (0.61; 0.7)	0.58–0.72	0.576
sCr [mg/dL] > 18 years old	0.6–1.1	0.76 (0.66; 0.85)	0.57–1	0.83 (0.74; 0.91)	0.59–1.02	0.196
sUrea [mg/dL]	19–49	27 (23; 32)	15–66	25 (22; 28)	17–34	0.097
eGFR [mL/min]	>90	106 (82.5; 117)	56–152	118 (107.5; 130)	89–130	0.002
eGFRcysC [mL/min]	>90	81 (64; 107)	41–125	99 (89; 108)	68–148	0.007
uNGAL [ng/mL]	0–131.7	40.83 (20.9; 56.3)	7.6–127	34.65 (20.1; 48.5)	10.5–92.5	0.766

* Abbreviations: CysC—serum Cystatin C; eGFR—estimated glomerular filtration rate based on creatinine; eGFRcysC—estimated glomerular filtration rate based on cystatin C; sCr—serum creatinine; sUrea—serum urea; uNGAL—urinary neutrophil gelatinase-associated lipocalin.

## Data Availability

Data supporting the results of this study shall, upon appropriate request, be available from the corresponding author.
